# Preclinical immune efficacy against SARS-CoV-2 beta B.1.351 variant by MVA-based vaccine candidates

**DOI:** 10.3389/fimmu.2023.1264323

**Published:** 2023-12-12

**Authors:** Patricia Pérez, Guillermo Albericio, David Astorgano, Sara Flores, Cristina Sánchez-Corzo, Pedro J. Sánchez-Cordón, Joanna Luczkowiak, Rafael Delgado, José M. Casasnovas, Mariano Esteban, Juan García-Arriaza

**Affiliations:** ^1^ Department of Molecular and Cellular Biology, Centro Nacional de Biotecnología (CNB), Consejo Superior de Investigaciones Científicas (CSIC), Madrid, Spain; ^2^ Centro de Investigación Biomédica en Red de Enfermedades Infecciosas (CIBERINFEC), Madrid, Spain; ^3^ Pathology Department, Centro de Investigación en Sanidad Animal (CISA), Instituto Nacional de Investigación y Tecnología Agraria y Alimentaria (INIA), Consejo Superior de Investigaciones Científicas (CSIC), Madrid, Spain; ^4^ Instituto de Investigación Hospital Universitario 12 de Octubre (imas12), Madrid, Spain; ^5^ Department of Medicine, School of Medicine, Universidad Complutense de Madrid, Madrid, Spain; ^6^ Department of Macromolecular Structures, Centro Nacional de Biotecnología (CNB), Consejo Superior de Investigaciones Científicas (CSIC), Madrid, Spain

**Keywords:** COVID-19, SARS-CoV-2, MVA-based vaccine, variants of concern, S protein, immunogenicity, efficacy, mice

## Abstract

The constant appearance of new severe acute respiratory syndrome coronavirus 2 (SARS-CoV-2) variants of concern (VoCs) has jeopardized the protective capacity of approved vaccines against coronavirus disease-19 (COVID-19). For this reason, the generation of new vaccine candidates adapted to the emerging VoCs is of special importance. Here, we developed an optimized COVID-19 vaccine candidate using the modified vaccinia virus Ankara (MVA) vector to express a full-length prefusion-stabilized SARS-CoV-2 spike (S) protein, containing 3 proline (3P) substitutions in the S protein derived from the beta (B.1.351) variant, termed MVA-S(3Pbeta). Preclinical evaluation of MVA-S(3Pbeta) in head-to-head comparison to the previously generated MVA-S(3P) vaccine candidate, expressing a full-length prefusion-stabilized Wuhan S protein (with also 3P substitutions), demonstrated that two intramuscular doses of both vaccine candidates fully protected transgenic K18-hACE2 mice from a lethal challenge with SARS-CoV-2 beta variant, reducing mRNA and infectious viral loads in the lungs and in bronchoalveolar lavages, decreasing lung histopathological lesions and levels of proinflammatory cytokines in the lungs. Vaccination also elicited high titers of anti-S Th1-biased IgGs and neutralizing antibodies against ancestral SARS-CoV-2 Wuhan strain and VoCs alpha, beta, gamma, delta, and omicron. In addition, similar systemic and local SARS-CoV-2 S-specific CD4^+^ and CD8^+^ T-cell immune responses were elicited by both vaccine candidates after a single intranasal immunization in C57BL/6 mice. These preclinical data support clinical evaluation of MVA-S(3Pbeta) and MVA-S(3P), to explore whether they can diversify and potentially increase recognition and protection of SARS-CoV-2 VoCs.

## Introduction

SARS-CoV-2 virus has spread worldwide since 2019, causing as of July 2023 more than 700 million cases and close to 7 million deaths. The fast development of vaccines has made it possible to prevent severe illness and minimize the risk of death. However, immediately after the outbreak, several variants of concern (VoCs) emerged, each containing numerous mutations within the viral genome (1). SARS-CoV-2 VoCs, with mutations in the spike (S) protein, specifically in the receptor-binding domain (RBD), spread more efficiently and escape to neutralization by antibodies induced by vaccination or infection (1–8). Among the VoCs, SARS-CoV-2 beta (B.1.351) variant is of special interest for this work. SARS-CoV-2 B.1.351 was first identified in October 2020 in South Africa and contains 10 amino acid substitutions in the S protein, with three of them in the RBD that are reported to increase binding between S and its cell receptor angiotensin converting enzyme 2 (ACE2), and also results in a reduced level of neutralization by natural and vaccine-induced antibodies (8, 9). Moreover, recently the European Medicines Agency (EMA) recently approved for human use a protein-based COVID-19 vaccine, termed Bimervax, consisting of a fusion RBD heterodimer of Wuhan and beta (B.1.351) variant strains administered with an adjuvant (https://www.ema.europa.eu/en/medicines/human/EPAR/bimervax) (10–12).

The continued emergence of SARS-CoV-2 VoCs has led to imminent challenges in establishing protective immunity by using approved first-generation COVID-19 vaccines (based in the ancestral Wuhan strain), as two or three doses of approved COVID-19 vaccines confer variable efficacy against various SARS-CoV-2 VoCs (13–22). Following the same line of results, different Wuhan-based COVID-19 vaccines have been reported to protect against morbidity and mortality due to SARS-CoV-2 VoCs in several animal models, although with distinct degrees of protection against infection (23–32). Although Wuhan-based COVID-19 vaccines have proven useful in decreasing the number of hospitalizations and deaths caused by VoCs, new variant-specific vaccines capable of increasing protection capacity against disease and viral transmission are desirable.

We have previously described that modified vaccinia virus Ankara (MVA) vectors expressing full-length native (MVA-S) or prefusion-stabilized Wuhan-derived SARS-CoV-2 S proteins (MVA-S(3P)) were highly immunogenic and effective in mice (33–38), hamsters (39) and rhesus macaques (40). In particular, MVA-S(3P) was more immunogenic than MVA-S, and a single dose induced antibodies that neutralized several VoCs and protected K18-hACE2 mice from a lethal challenge with the ancestral Wuhan SARS-CoV-2 (isolate MAD6, containing the mutation D614G in the S protein) (36, 37). Moreover, we have recently reported that MVA-S(3P) fully protects against SARS-CoV-2 infection in hamsters (41). Here, to extend our previous studies on the capacity of MVA-based vaccine candidates to prevent SARS-CoV-2 infections, we describe the generation and characterization of an optimized MVA-based vaccine candidate expressing a human codon optimized full-length prefusion-stabilized S protein derived from SARS-CoV-2 beta (B.1.351). Head-to-head comparison of the immunogenicity and efficacy of MVA-S(3Pbeta) and MVA-S(3P) in C57BL/6 and transgenic K18-hACE2 mice, respectively, revealed that both vaccine candidates elicited potent and similar T-cellular and humoral immune responses against ancestral SARS-CoV-2 Wuhan strain and beta (B.1.351) variant and cross-neutralizing antibodies against other VoCs of human health relevance. Importantly, both vaccine candidates similarly protected K18-hACE2 mice from a lethal challenge with beta (B.1.351) variant, significantly reducing mRNA and infectious viral loads in the lungs and broncoalveolar lavages (BAL), pulmonary histopathological lesions, and levels of proinflammatory cytokines in the lungs. These findings highlight the importance of defining the preclinical immune efficacy against emerging VoCs of Wuhan- and VoC-based vaccines, to assure activation of markers of protection.

## Materials and methods

### Animals and ethics statement

Female C57BL/6OlaHsd mice (6–8 weeks old) used for immunogenicity experiments were purchased from Envigo Laboratories and stored in the animal facility of the Centro Nacional de Biotecnología (CNB) (Madrid, Spain). Female mice of the transgenic K18-hACE2 line, which express the human ACE2 receptor, were acquired from the Jackson Laboratory [034860-B6.Cg-Tg(K18-ACE2)2Prlmn/J, genetic background C57BL/6J x SJL/J)F2], and efficacy experiments using these mice were performed in the biosafety level 3 (BSL-3) facilities at the Centro de Investigación en Sanidad Animal (CISA)-Instituto Nacional de Investigaciones Agrarias (INIA-CSIC) (Valdeolmos, Madrid, Spain). The Ethical Committees of Animal Experimentation (CEEA) of the CNB-CSIC, CISA-INIA-CSIC and the Division of Animal Protection of the Comunidad de Madrid approved these animal studies (PROEX 49/20, 169.4/20 and 161.5/20). Animal procedures followed the international guidelines and Spanish law under the Royal Decree (RD) 53/2013.

### Cells

DF-1 cells (a spontaneously immortalized chicken embryo fibroblast [CEF] cell line; ATCC catalog no. CRL-12203) and HeLa cells (human epithelial cervix adenocarcinoma; ATCC catalog no. CCL-2) were cultured in Dulbecco’s modified Eagle’s medium (DMEM) (Gibco-Life Technologies) supplemented with penicillin (100 U/mL; Sigma-Aldrich), streptomycin (100 mg/mL; Sigma-Aldrich), L-glutamine (2 mM; Sigma-Aldrich), non-essential amino acids (0.1 mM; Sigma-Aldrich), amphotericin B (Fungizone, 0.5 mg/mL; Gibco-Life Technologies), gentamicin (50 mg/mL; Sigma-Aldrich) and 10% heat-inactivated fetal bovine serum (FBS) (Gibco-Life Technologies). Vero-E6 cells (from African green monkey kidney, ATCC catalog no. CRL-1586) were grown in DMEM (Gibco-Life Technologies) supplemented with HEPES (10 mM; Gibco-Life Technologies), non-essential amino acids (0.1 mM; Sigma-Aldrich), penicillin (100 U/mL; Sigma-Aldrich), streptomycin (100 mg/mL; Sigma-Aldrich), and 10% heat inactivated FBS. Vero/TMPRSS2 (VeroE6 cell line modified to constitutively express TMPRSS2 serine protease, under geneticin selection, in order to be highly susceptible to SARS-CoV-2 infection) were maintained in DMEM (Gibco-Life Technologies) supplemented with HEPES (10 mM; Gibco-Life Technologies), nonessential amino acids (0.1 mM; Sigma-Aldrich), penicillin (100 U/mL; Sigma-Aldrich), streptomycin (100 mg/mL; Sigma-Aldrich), Geneticin (G418, 1 mg/mL, Merck-Life Sciences), and 10% heat inactivated FBS. Cell cultures were maintained at 37°C in a humidified incubator containing 5% CO_2_.

### Viruses

We used the attenuated MVA-WT poxvirus strain, derived from the Chorioallantois vaccinia virus Ankara strain (42) and the MVA-S(3P) vaccine candidate (36, 37). MVA-WT was employed as the parental virus for the generation of the MVA-S(3Pbeta) vaccine candidate expressing a beta (B.1.351) derived human codon optimized full-length prefusion-stabilized SARS-CoV-2 S protein, containing three mutations in the furin cleavage site (R682G, R683S, and R685S) to prevent cleavage of the S protein in S1 and S2 domains, and 3 proline (3P) substitutions in the S2 region that stabilize the S protein in a prefusion conformation (A942P, K986P, and V987P). All MVA viruses were grown in permissive culture chicken cells (DF-1) to produce a master virus seed stock (passage 2 [P2] stock) and titrated in DF-1 cells using a plaque immunostaining assay, as previously described (43). For use as inoculum in animal experiments, MVA viruses were expanded in DF-1 cells and purified by centrifugation through two 36% (wt/vol) sucrose cushions in 10 mM Tris-HCl (pH 9). All viral stocks were free of contamination with mycoplasma (checked by Mycoplasma Gel Detection kit; Biotools), bacteria (checked by growth on LB plates without ampicillin), or fungi (checked by growth on Columbia blood agar plates; Oxoid).

The SARS-CoV-2 beta (B.1.351) variant (hCoV-19/France/PDL-IPP01065i/2021), was supplied through the European Virus Archive-Global (Evag) platform, a project funded by the European Union’s Horizon 2020 research and innovation programme under grant agreement No. 653316. The virus was sent by the National Reference Centre for Respiratory Viruses hosted by Institut Pasteur (Paris, France) headed by Pr. Sylvie van der Werf, and the human sample from which the virus was isolated by Dr. Besson J., Bioliance Laboratory, Saint-Herblain; France. The stock virus was amplified by propagation in Vero-TMPRSS2 cells by inoculation at a multiplicity of infection (MOI) of 0.001 plaque-forming units (PFUs)/cell (passage 2). Cell supernatants were harvested at 72 h post-infection (hpi), cleared by centrifugation, aliquoted, and stored at −80°C. Virus infectivity titers were determined by standard plaque assay in Vero-E6 cells or by median tissue culture infectious dose (TCID_50_) assays in Vero-TMPRSS2 cells. The full-length viral genome was sequenced, and found to be identical to SARS-CoV-2 B.1.351 reference sequence (hCoV-19/South Africa/N00344/2020; GISAID accession ID: EPI_ISL_712096).

Finally, we have also used SARS-CoV-2 strain MAD6 (kindly provided by José M. Honrubia and Luis Enjuanes, CNB-CSIC, Madrid, Spain) (44) that is similar to the Wuhan strain but with the D614G mutation in the S protein. The stock virus was prepared as previously described (37).

### Construction of plasmid transfer vector pCyA-S(3Pbeta) and generation of MVA-S(3Pbeta) vaccine candidate

The plasmid transfer vector pCyA-S(3Pbeta) was designed to generate the MVA-S(3Pbeta) vaccine candidate. A human codon optimized full-length prefusion-stabilized SARS-CoV-2 beta (B.1.351) strain S gene (hCoV-19/South Africa/N00344/2020; GISAID accession ID: EPI_ISL_712096) was inserted into the thymidine kinase (TK) locus of the parental virus MVA-WT, under the transcriptional control of the viral synthetic early/late (sE/L) promoter and with a Kozak sequence (GCCACC) before the ATG initiation codon of the S gene. Briefly, a 3,822-kbp DNA fragment encoding a human codon optimized SARS-CoV-2 full-length prefusion-stabilized beta (B.1.351) S gene was synthesized by GeneArt and inserted into plasmid vector pCyA (45), obtaining the plasmid transfer vector pCyA-S(3Pbeta) (11,322 bp). This plasmid includes a β-galactosidase (β-Gal) reporter gene between two repetitions of the left TK-flanking arm, which allows the deletion by homologous recombination of the reporter from the final recombinant virus after successive passages. The encoded human codon optimized full-length beta (B.1.531) S protein also contains 3 mutations in the furin cleavage site (R682G, R683S, and R685S) to prevent cleavage of the S protein in S1 and S2 domains, and the same 3 proline (3P) stabilizing amino acid mutations (A942P, K986P, and V987P) included in the MVA-S(3P) vaccine candidate validated previously (37).

Cultured DF-1 cells (3 × 10^6^ cells) were infected with parental MVA-WT virus at a MOI of 0.02 PFUs/cell and transfected 1 h later with 10 μg of DNA plasmid pCyA-S(3Pbeta), using Lipofectamine 2000 (Invitrogen) reagent, according to the manufacturer’s recommendations. At 72 hpi, cells were harvested, lysed by freeze–thaw cycling, sonicated, and used for recombinant virus screening. Recombinant MVA-S(3Pbeta) viruses containing the SARS-CoV-2 beta (B.1.351) full-length prefusion-stabilized S gene, inserted in the TK locus, and transiently coexpressing the β-Gal marker gene were selected by three consecutive rounds of plaque purification in DF-1 cells stained with X-Gal (5-bromo-4-chloro-3-indolyl-β-D-galactopyranoside, 1.2 mg/mL) (Sigma-Aldrich). In subsequent plaque purification steps, recombinant MVA-S(3Pbeta) viruses with the β-Gal gene deleted by homologous recombination between the left TK arm and the short-left TK arm repeat flanking the marker were isolated by three additional consecutive rounds of plaque purification screening for non-staining viral foci in DF-1 cells in the presence of X-Gal (1.2 mg/mL). In each round of plaque purification, the isolated plaques were grown in DF-1 cells, and the crude viruses obtained were used for the next round of plaque purification. The resulting recombinant virus MVA-S(3Pbeta) was grown, purified and titrated as previously described (37).

### Expression of SARS-CoV-2 S protein by western blotting and analysis of virus growth

To check the correct expression of SARS-CoV-2 prefusion-stabilized S protein by MVA-S(3P) and MVA-S(3Pbeta) vaccine candidates, monolayers of HeLa cells grown in 24-well plates were infected at 5 PFUs/cell with MVA-S(3P) and MVA-S(3Pbeta) (or with control virus MVA-WT). At different times (4 or 24 hpi), equal amounts of cell extracts were solubilized under reducing conditions (in the presence of 1× Laemmli plus β-mercaptoethanol). The Wester Blotting conditions employed were already detailed (37). We evaluated the expression of SARS-CoV-2 S protein with a rabbit polyclonal anti-SARS-CoV-2 S antibody (Genetex; recognizing SARS-CoV-2 S1 region). For a viral loading control, we used a rabbit anti-VACV E3 (CNB) antibody. An anti-rabbit HRP-conjugated antibody (Sigma-Aldrich) was used as the secondary antibody. The genetic stability of MVA-S(3Pbeta) vaccine candidate was also analysed by Wester Blotting. We confirmed the expression of SARS-CoV-2 S protein during 9 consecutive passages as previously reported (37).

Besides, we studied the virus growth profile of MVA-S(3P) and MVA-S(3Pbeta) analyzing viral titers after different times of infection (0, 24, 48, 72 hpi) as previously described for the validation of other vaccine candidates (34).

### Efficacy study schedule in K18-hACE2 transgenic mice

Female K18-hACE2 mice (9 weeks old at the beginning of the study) immunized with two intramuscular (IM) doses of MVA-S(3P) or MVA-S(3Pbeta) were used to evaluate the efficacy of the vaccine candidates. Groups of animals (n = 11) received two doses, with a 4-week interval (days 0 and 27), of 1 x 10^7^ PFUs of MVA-S(3P) or MVA-S(3Pbeta) by IM route in 100 μL of PBS (50 μL/leg). Mice inoculated with non-recombinant MVA-WT were used as a control group. On days 14 post-prime and 21 post-boost (day 48), blood was collected from each mouse by submandibular bleeding. The blood was incubated at 37 °C for 1 h, maintained at 4 °C overnight, and centrifuged at 3,600 rpm for 20 min at 4 °C to obtain serum samples. The obtained serum samples were then inactivated at 56 °C for 30 min and stored at -20 °C until analysis of humoral immune responses. Four weeks after the second immunization (day 56), all mice were anesthetized in an isoflurane chamber and challenged with a lethal dose (1 x 10^5^ PFUs) of SARS-CoV-2 beta (B.1.351) strain by the intranasal (IN) route in 50 μL of PBS, after being anesthetized in an isoflurane chamber. Mice were then monitored for body weight changes, signs and symptoms of disease and mortality for 10 days post-challenge. Animals with more than a 20% of weight loss or presenting severe signs and symptoms of disease (lack of movement, breathing difficulties, etc) were euthanized by cervical dislocation. At 4 days post-challenge (day 60), at least three mice per group were euthanized, and lung, BAL, and serum samples were collected. The entire left lung lobe was removed from each mouse and immersion-fixed in zinc formalin (Sigma-Aldrich) for 48 h. After the fixation period, samples were routinely processed and embedded in paraffin for subsequent histopathological evaluation. The right lung lobes were divided longitudinally into two, with one part placed in RNALater stabilization reagent (Sigma-Aldrich) and stored at -80°C until RNA extraction, and the other lung part was weighed and stored at -80 °C until analysis of virus yields. BAL from each mouse was collected by flushing into the trachea 700 μL of Roswell Park Memorial Institute (RPMI) 1640 medium (Gibco-Life Technologies) supplemented with HEPES (10 mM; Gibco-Life Technologies), β-mercaptoethanol (10 µM; Sigma-Aldrich), and L-glutamine (2 mM; Merck); then, the samples were spun down to remove cellular debris, and supernatants were stored at -80°C until viral load detection and RNA extraction. Blood was collected and processed to obtain serum samples following the procedure previously described after the immunization and prechallenge.

### Quantification of SARS-CoV-2 mRNA and cytokine mRNA by reverse transcription-quantitative polymerase chain reaction

Lung and BAL samples obtained from K18-hACE2 mice on day 4 post-challenge. Lungs were stored in RNALater (Sigma-Aldrich) at -80°C, were homogenized using a gentleMACS dissociator (Miltenyi Biotec) in 2 mL of RLT buffer (Qiagen) containing β-mercaptoethanol (Sigma-Aldrich). A total of 600 μL of homogenized lung tissue was utilized to isolate total RNA using the RNeasy Mini Kit (Qiagen), according to the manufacturer’s specifications. On the other hand, 50 μL of BAL samples were used to extract RNA using an in-house TRIzol® (Invitrogen) method, following a protocol described elsewhere (46).

Lung and BAL were subjected to analysis for quantifying SARS-CoV-2 mRNA using RT-qPCR, following a previously described method (36, 37). SARS-CoV-2 viral mRNA content was determined using a previously validated set of primers and probes specific for the SARS-CoV-2 subgenomic RNA for protein E and the genomic virus RNA-dependent RNA polymerase (RdRp) gene, and gene expression was normalized to the expression of the cellular 28S ribosomal RNA gene (47). Additionally, the mRNA expression levels of key proinflammatory cytokines (IL-6, IL-12b, CCL2, CCL12, IFNb1, TNF-α and CXCL10) were also measured in lung samples using specific TaqMan probes (Thermo Fisher Scientific; the sequence will be provided upon request). The specific gene expression was also presented relative to the expression of the cellular 28S ribosomal RNA gene, as previously described (36, 37). mRNA arbitrary units (A.U.) were quantified relative to negative RNA samples (from uninfected mice) using the 2^-ΔΔCt^ method. All samples were tested in duplicates.

### Analysis of SARS-CoV-2 virus yields by plaque assay

Lung and BAL samples from K18-hACE2 mice, harvested at day 4 post-challenge, were analyzed for the presence of SARS-CoV-2 infectious virus using a plaque assay, as previously described (36, 37). Lungs were harvested, weighed, and stored directly at -80°C until homogenization with a gentleMACS dissociator (Miltenyi Biotec) in 2 mL of PBS buffer. Undiluted and serial ten-fold dilutions of homogenized lung tissue or BAL samples were added in triplicate to Vero-E6 cell monolayers seeded in 12-well plates at 5 x 10^5^ cells/well. After 1 h of adsorption, the inoculum was removed and plates were incubated at 37°C, 5% CO_2_ in 2:1 DMEM 2X-4% FBS : Avicel® RC-591 (microcrystalline cellulose and carboxymethylcellulose sodium, DuPont Nutrition Biosciences ApS). After 4 days, cells were fixed for 1 h with 10% formaldehyde (Sigma-Aldrich), the supernatant was removed, and plaques were visualized by adding 0.5% crystal violet (Sigma-Aldrich). SARS-CoV-2 titers were determined in PFUs per gram of lung tissue or in PFUs per mL of BAL.

### Lung histopathology

Lung histopathology was performed as previously described (36, 37). To assess the character and severity of histopathological lesions, lung inflammation scoring parameters based on previous reports on SARS-CoV-2 infection in mouse models were used (48). These histopathological parameters were graded following a semi-quantitative scoring system as follows: (0) no lesion; (1) minimal lesion; (2) mild lesion; (3) moderate lesion; (4) severe lesion. The cumulative scores of the histopathological lesions provided the total score for each animal. In each experimental group, individual scores were used to calculate the group average. In addition, H&E-stained sections were visually scored 0–6 based on the percentage of lung area affected by inflammatory lesions as follows: 0% lung injury (score 0); < 5% (score 1); 6-10% (score 2); 11–20% (score 3); 21–30% (score 4); 31–40% (score 5); > 40% (score 6). In each experimental group, individual scores were used to calculate the group average.

### Enzyme-linked immunosorbent assay

The titers of binding anti-S IgG, IgG1, IgG2c and IgG3 antibodies in individual or pooled sera from immunized mice were measured by ELISA, as previously described (36, 37). Total binding anti-S IgG endpoint titers were measured as the last serum dilution that gave an absorbance value at 450 nm at least three times higher than that of a naive serum. The soluble SARS-CoV-2 S proteins used to coat the plates were derived from the Wuhan strain (GenBank accession number MN908947.3) or the beta (B.1.351) variant (GISAID: EPI_ISL_712096) and the recombinant expression vectors were prepared as previously described (36, 37). The soluble S proteins were expressed in mammalian cells and purified from cell supernatants as reported (34); they were used to analyze the levels of IgG antibodies in mice serum samples by ELISA.

### Neutralization of live SARS-CoV-2 or pseudotyped variants of concern

The capacity of the sera obtained from immunized mice to neutralize live SARS-CoV-2 virus was measured using a microneutralization test (MNT) assay in a BSL-3 laboratory at the CNB-CSIC, as previously described (36, 37). Serially diluted serum samples in DMEM-2% FBS medium were incubated at a 1:1 ratio with 200 TCID50 of SARS-CoV-2 parental Wuhan strain virus (MAD6 isolate, containing the D614G mutation in the S protein) or beta (B.1.351) variant in 96-well tissue culture plates for 1 h at 37°C. Then, mixtures of serum samples and SARS-CoV-2 were added in triplicate to Vero-TMPRSS2 cell monolayers seeded in 96-well plates at 2 x 10^4^ cells/well. To obtain the 50% neutralization titers (NT50), half maximal effective concentration (EC50) and 95% confidence intervals (95% CI) were calculated using a nonlinear regression model fit with settings for agonist concentration versus normalized response curve using GraphPad Prism v9 Software.

The capacity of pooled serum samples obtained from K18-hACE2 immunized mice to neutralize different SARS-CoV-2 VoCs was tested using SARS-CoV-2 pseudotyped vesicular stomatitis virus (VSV) expressing the SARS-CoV-2 S protein, as previously described (36, 37). The SARS-CoV-2 S variants used were S_614G, alpha (B.1.1.7), beta (B.1.351), gamma (P.1), delta (B.1.617.2), and omicron (BA.4/BA.5), and were produced as described elsewhere (49). The SARS-CoV-2 S mutant D614G was generated by site-directed mutagenesis (Q5 Site Directed Mutagenesis Kit; New England Biolabs) following the manufacturer’s instructions and used as an input DNA pcDNA3.1 expression vector encoding SARS-CoV-2 S_614D (34). The SARS-CoV-2 alpha (B.1.1.7; GISAID: EPI_ISL_608430), beta (B.1.351; GISAID: EPI_ISL_712096), gamma (P.1; GISAID: EPI_ISL_833140), delta (B.1.617.2; GISAID: EPI_ISL_1970335), and omicron (BA.4/BA.5; GISAID: EPI_ISL_13424827) VoCs were optimized, synthesized, and cloned into pcDNA3.1 by GeneArt (Thermo Fisher Scientific, GeneArt GmbH, Regensburg, Germany). NT_50_ titers, EC50 and 95% CI were calculated using a nonlinear regression model fit with settings for log(agonist concentration) versus normalized response curve using GraphPad Prism v9 Software.

### Immunogenicity study schedule in C57BL/6 mice

To evaluate the immunogenicity of the MVA-based vaccine candidates against COVID-19, groups of female C57BL/6 mice (n = 6 per group; 6 to 8 weeks old) were slightly anesthetized with isoflurane (1-chloro-2,2,2-trifluoroethyl difluoromethyl ether; Isoflo®, Zoetis Belgium SA) and each mouse received one dose of 1 × 10^7^ PFUs of MVA-S(3P) or MVA-S(3Pbeta) by the IN route in 50 μl of PBS. Mice inoculated with nonrecombinant MVA-WT were used as a control group. No adverse effects were detected in immunized mice. Then, 14 days after the immunization, mice were euthanized by using a lethal dose of 10% xylazine (Xilagesic 20 mg/mL; Laboratorios Calier, Barcelona, Spain) + 10% ketamine (Imalgene 100 mg/mL; Merial Laboratorios, Barcelona, Spain). Blood from each mouse was collected by cardiac puncture, maintained at 37°C for 1 h, kept at 4°C overnight, and centrifuged at 3,600 rpm for 20 min at 4°C to obtain serum samples that were stored at −20°C until used, to analyze SARS-CoV-2-specific humoral immune responses. Spleens, and bronchial lymph nodes (BLNs) extracted from each mouse were pooled per group, processed mechanically, blood-cell depleted, and filtered through 40-µm cell strainers until single-cell samples were obtained, which were used to measure the SARS-CoV-2 S-specific T-cell immune responses by an intracellular cytokine staining (ICS) assay.

### ICS assay

The magnitude of SARS-CoV-2 S-specific CD4^+^ and CD8^+^ T cells expressing CD107a, and/or secreting IFNγ, and/or TNFα, and/or IL-2 were analyzed by an ICS assay as previously described (34) in cells (splenocytes or BLN cells) stimulated with two SARS-CoV-2 S peptide pools (1 µg/mL) (JPT Peptide Technologies, Berlin, Germany), spanning the S1 and S2 regions as consecutive 15-mers overlapping by 11 amino acids of the S protein from the Wuhan strain or beta variant. Moreover, the magnitude of SARS-CoV-2 S-specific CD4^+^ T follicular helper (Tfh) cells expressing CD154, and/or secreting IFNγ, and/or IL-21 were also analyzed by an ICS assay as previously described (34) in splenocytes stimulated with a matching SARS-CoV-2 S protein (5 µg/mL) plus S1 and S2 peptide pools (1 µg/mL). Cells were acquired with a Gallios flow cytometer (Beckman Coulter), and analyses of the data were performed with the FlowJo software version 10.4.2 (Tree Star), as previously described (34).

### Statistical procedures

All graphs, calculations, and statistical analyses were performed using GraphPad Prism software version 9.4.1 (GraphPad Software). To determine differences between groups, ordinary one-way ANOVA of transformed data followed by Tukey’s multiple comparison test was used for the statistical analysis of SARS-CoV-2 and cytokine mRNA levels, and SARS-CoV-2 virus yields. An unpaired nonparametric Mann-Whitney test was employed for the statistical evaluation of lung histopathological scores and an ordinary one-way ANOVA followed by Tukey’s multiple comparison test for the percentage of lung area with lesions. An unpaired nonparametric Mann-Whitney test of transformed data was used for statistical analysis between groups of IgG titers, an unpaired t-test of transformed data for the live virus NT_50_ neutralizing titers, and an unpaired t-test with Welch’s correction of transformed data for NT_50_ neutralizing titers of pooled mouse serum samples against SARS-CoV-2 pseudotyped VSVs. Statistical analysis of the ICS assay data was realized as previously described (50), using an approach that corrects measurements for background response, calculating confidence intervals and p-values. Statistical significance is indicated as follows: *p < 0.033; **p < 0.002; ***p < 0.0002; ****p<0.0001.

## Results

### Generation of an MVA-based vaccine candidate expressing a prefusion-stabilized S protein from the SARS-CoV-2 beta (B.1.351) variant of concern

**Figure 1:**
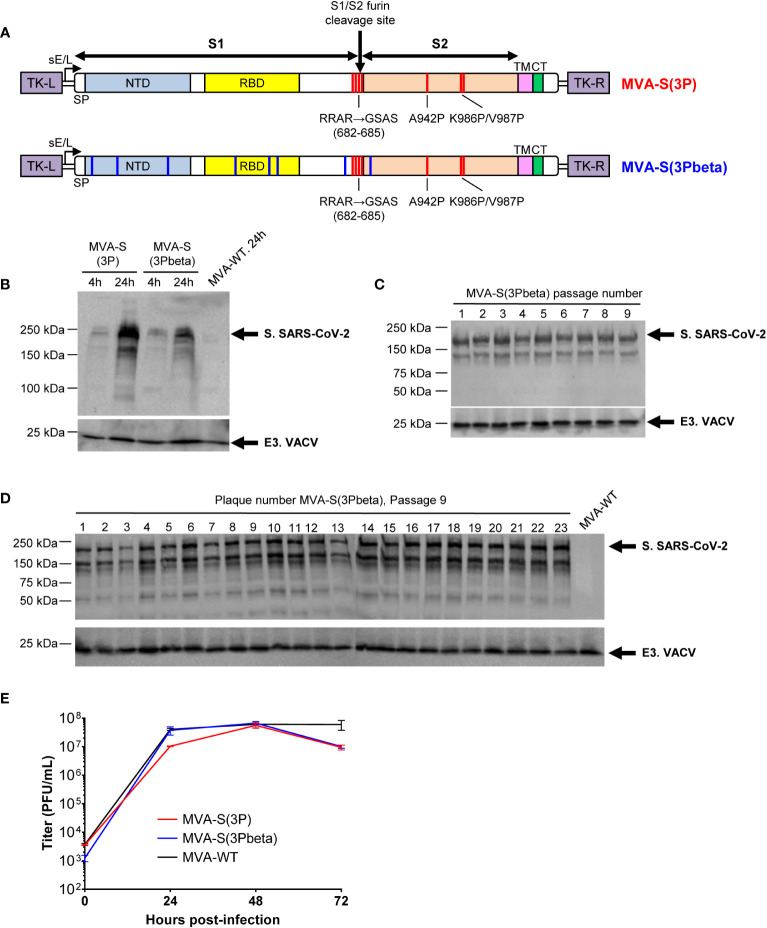
Design, generation, and *in vitro* characterization of MVA-S(3Pbeta) vaccine candidate. **(A)** Scheme of the prefusion-stabilized full-length S proteins inserted in the MVA genome to generate the MVA-S(3P) and MVA-S(3Pbeta) vaccine candidate. S1 and S2 regions are indicated, together with the amino acid mutations in the furin cleavage site and changes to prolines in the S2 region (indicated in red). The amino acid mutations of beta (B.1.351) variant are also indicated in blue. The SARS-CoV-2 S gene is inserted within the TK locus of MVA-WT virus and is driven by the sE/L virus promoter. NTD, N-terminal domain; RBD, receptor binding domain; TM, transmembrane; CT, cytoplasmic tail; TK-L, TK left; TK-R, TK right. **(B)** Expression of SARS-CoV-2 S protein by MVA-S(3P) and MVA-S(3Pbeta) vaccine candidates. Western blotting of MVA-infected (5 PFUs/cell) HeLa cell extracts at 4 and 24 hpi. Rabbit polyclonal anti-S and anti-VACV E3 antibodies were used for protein identification on 7% SDS-PAGE under reducing conditions. Size (in kilodaltons [kDa]) and migration of molecular weight markers are indicated. **(C, D)** Genetic stability of MVA-S(3Pbeta) vaccine candidate. Western blotting of DF-1 cell samples (24 hpi) infected with initial P2 stock and with 9 successive passages of MVA-S(3Pbeta) viruses **(C)** or from 23 individual virus plaques picked after 9 consecutive cell infection cycles **(D)**. Samples were analyzed under reducing conditions. Rabbit polyclonal anti-S and anti-VACV E3 antibodies were used for protein identification. **(E)** Viral growth kinetics of MVA-S(3Pbeta). Monolayers of DF-1 cells were infected at 0.01 PFUs/cell with MVA-WT, MVA-S(3P) or MVA-S(3Pbeta). At different times postinfection (0, 24, 48, and 72 hpi), virus titers in cell lysates were quantified by a plaque immunostaining assay. The means of results from two independent experiments are shown.

We previously reported the generation and preclinical evaluation of the immunogenicity and efficacy of an MVA-based vaccine candidate, named MVA-S(3P), which expressed a full-length prefusion-stabilized S protein from the ancestral SARS-CoV-2 Wuhan strain (36, 37). The S protein was stabilized in the prefusion conformation by introducing three proline substitutions in the S2 region (A942P, K986P, and V987P) and the furin cleavage site was mutated to avoid the processing of S into the S1 and S2 regions and fusion activation (**Figure 1A**). When compared to an MVA vector expressing a non-stabilized S protein (MVA-S), MVA-S(3P) increased the levels of expression of the S protein by 2-fold, induced higher binding IgG and neutralizing antibody titers against SARS-CoV-2 Wuhan strain and several VoCs and a single dose of the vaccine better protected mice against a lethal challenge with the ancestral SARS-CoV-2 Wuhan strain (36, 37). Now, following the identification of the SARS-CoV-2 beta (B.1.351) variant (9), we successfully generated a new MVA-based vaccine candidate expressing a full-length prefusion-stabilized S protein from the beta (B.1.351) variant, termed MVA-S(3Pbeta), which also contains three proline substitutions in the S2 region (A942P, K986P, and V987P) and the furin cleavage site mutated (**Figure 1A**). The expression profile of the S protein was characterized at different time points in non-permissive human HeLa cells infected with MVA-S(3P) and MVA-S(3Pbeta), revealing a major 180-kDa protein product (**Figure 1B**). The analysis by Western blot of SARS-CoV-2 S protein expression during successive passages in permissive DF-1 cell cultures infected at low MOI (0.05 PFUs/cell) with MVA-S(3Pbeta) showed that MVA-S(3Pbeta) efficiently expressed the S protein during 9 successive passages, demonstrating a high genetic stability (**Figure 1C**). In addition, stable S protein expression was determined in all individual plaques isolated at passage 9 (**Figure 1D**). Finally, the evaluation in permissive DF-1 cells of the growth kinetics of MVA-S(3Pbeta), compared with MVA-S(3P) and MVA-WT, showed that all viruses had a comparable growth kinetics (**Figure 1E**).

### MVA-S(3Pbeta) and MVA-S(3P) fully prevented morbidity and mortality in K18-hACE2 transgenic mice challenged with SARS-CoV-2 beta (B.1.351), reducing SARS-CoV-2 virus replication, lung pathology, and levels of pro-inflammatory cytokines

**Figure 2:**
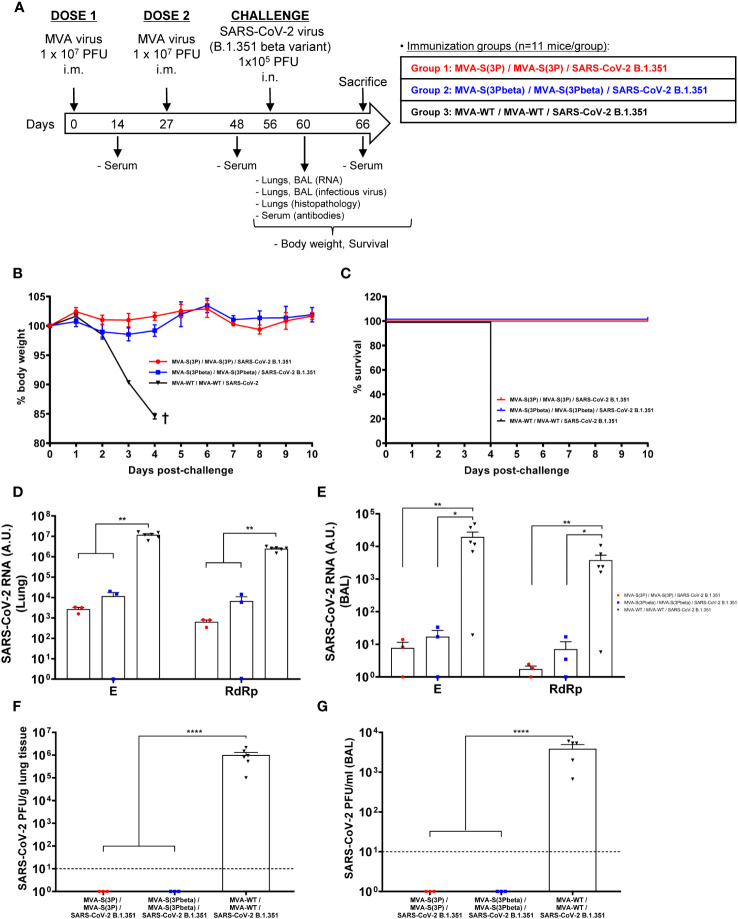
MVA-S(3P) and MVA-S(3Pbeta) vaccine candidates protects K18-hACE2 transgenic mice from SARS-CoV-2 beta (B.1.351) infection. **(A)** Efficacy schedule. Female K18-hACE2 transgenic mice (n=11 per group) were immunized by the IM route with two doses, spanned by 4 weeks, of 1 x 10^7^ PFUs of MVA-S(3P), MVA-S(3Pbeta) or MVA-WT as indicated. At day 14 post-prime and 21 post-boost, serum samples were obtained from each mouse, as indicated. At day 56 (4 weeks post-boost) mice were challenged intranasally with 1 x 10^5^ PFUs of SARS-CoV-2 beta variant (B.1.351). At day 4 postchallenge, at least 3 mice per group were sacrificed and lungs, BAL and serum samples collected as indicated. Serum was also collected at day 10 postchallenge in groups 1, 2 (in group 3 all mice have died by this day). **(B, C)** The challenged mice were monitored for change of body weight **(B)** and mortality **(C)** for 10 days. †: mice were euthanized due to loss of more than 20% of initial body weight. **(D, E)** Virus replication in lung samples **(D)** and BAL **(E)**. SARS-CoV-2 subgenomic E and genomic RdRp mRNA detected by RT-qPCR at 4 days after virus infection. Mean RNA levels (in arbitrary units [A.U.] normalized to uninfected mice) from duplicates of each lung and BAL samples and SEM of each group are represented. **(F, G)** SARS-CoV-2 infectious virus in lung samples **(F)** and BAL **(G)**. Mean (PFUs/g of lung tissue or PFUs/mL of BAL) from triplicates of each sample and SEM of each group are represented. The dashed line represents the limit of detection. Ordinary one-way ANOVA of transformed data followed by Tukey’s multiple comparison test: *p < 0.033; **p < 0.002; ****p<0.0001.

The efficacy triggered by MVA-S(3Pbeta) against SARS-CoV-2 beta (B.1.351) variant was then evaluated in transgenic K18-hACE2 mice (n = 11/group), susceptible to SARS-CoV-2 infection (51, 52), and compared to MVA-S(3P). Thus, mice were intramuscularly immunized with two doses of MVA-S(3Pbeta) or MVA-S(3P) (1 x 10^7^ PFUs/mouse) with a 4-week interval, and challenged 4 weeks later with a lethal dose (1 x 10^5^ PFUs/mouse) of SARS-CoV-2 beta (B.1.351) variant by the IN route (**Figure 2A**). Mice inoculated with MVA-WT were used as a control group of vector virus infection. Mice were supervised for changes in body weight and mortality for 10 days after SARS-CoV-2 infection. None of the mice immunized with MVA-S(3P) or MVA-S(3Pbeta) experience any body weight loss (**Figure 2B**) and exhibited 100% survival (**Figure 2C**). In contrast, mice immunized with MVA-WT showed significant body weight loss (more than 25%) (**Figure 2B**), and all mice died or were euthanized by day 4 post-challenge (**Figure 2C**).

To evaluate the effect of vaccination on SARS-CoV-2 replication, three mice per vaccinated group (n=6 in the MVA-WT control group) were sacrificed on day 4 after SARS-CoV-2 challenge, and lungs and BAL were collected and processed for the presence of SARS-CoV-2 subgenomic E and genomic RdRp RNA (**Figures 2D, E**), as well as for live infectious virus yields (**Figures 2F, G**). Subgenomic and genomic SARS-CoV-2 RNA levels were significantly lower in the lungs and BAL samples of mice immunized with MVA-S(3P) or MVA-S(3Pbeta) than in the control group immunized with MVA-WT (**Figures 2D, E**, respectively), with no significant differences between MVA-S(3P) and MVA-S(3Pbeta). Remarkably, no live infectious virus was detected in either the lungs or BAL samples of mice immunized with MVA-S(3P) or MVA-S(3Pbeta), whereas high titers of live virus were detected in the lungs and BAL samples of the MVA-WT control group (**Figures 2F, G**).

**Figure 3:**
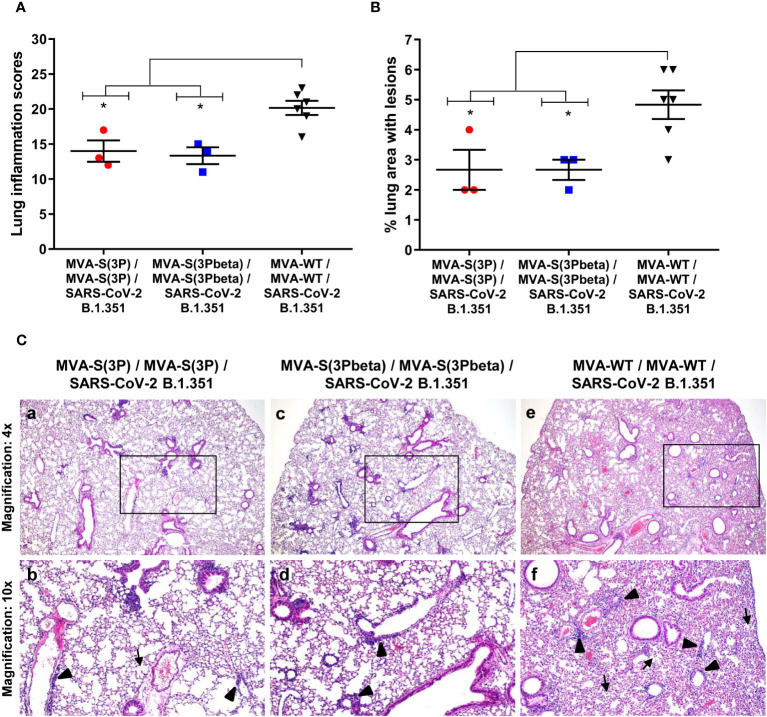
MVA-S(3P) and MVA-S(3Pbeta) vaccine candidates reduced SARS-CoV-2 beta variant lung pathology in K18-hACE2 transgenic mice. **(A)** Mean and SEM of cumulative histopathological lesion scores in lung samples taken from immunized K18-hACE2 mice that were euthanized at day 4 post-challenge. Unpaired nonparametric Mann-Whitney test: *p < 0.033. **(B)** Percentage of lung area affected by inflammatory lesions in lung samples taken from immunized K18-hACE2 mice that were euthanized at day 4 post-challenge. Ordinary one-way ANOVA followed by Tukey’s multiple comparison test: *p < 0.033. **(C)** Representative lung histopathological sections (H&E staining) observed in immunized K18-hACE2 transgenic mice that were euthanized at day 4 post-challenge. A general view of the lung area (magnification: 4x) along with histopathological details from selected lung areas (black boxes) have been displayed (magnification: 10x). In mice that were immunized with two doses of MVA-S(3P) and MVA-S(3Pbeta) (a, c), alveolar spaces were larger and evident while inflammatory changes were less severe than those observed in mice immunized with two doses of MVA-WT (e). Mice immunized with MVA-S(3P) and MVA-S(3Pbeta) showed mild lung lesions that were characterized by the presence of small perivascular and peribronchiolar mononuclear infiltrates mainly constituted by lymphocytes (arrowheads), as well as occasional alveoli with some scarce infiltrated mononuclear cells (arrows) (b, d). On the contrary, in mice immunized with MVA-WT (control non-protected group) inflammatory lesions were more severe and diffuse. Such lesions were characterized by the presence of numerous large and perivascular and peribronchiolar infiltrates (arrowheads), alveolar spaces densely populated by inflammatory cells (arrows) and generalized alveolar septa thickening (f).

Lung histopathological analysis at 4 days post-challenge (n=3/vaccinated group; n=6/MVA-WT control group) showed that mice vaccinated with MVA-S(3P) or MVA-S(3Pbeta) displayed lesser lung inflammation scores (**Figure 3A**) and minor percentages of lung areas with lesions (**Figure 3B**) than control MVA-WT mice. Illustrative images of lung sections are shown in **Figure 3C**; mice vaccinated with MVA-S(3P) or MVA-S(3Pbeta) only exhibited focal thickening of the alveolar septa, and sporadic occurrence of inflammatory cells within the alveoli. However, mice immunized with control MVA-WT displayed severe diffuse thickening of the alveolar septa, a higher presence of mononuclear cell infiltrates within alveolar spaces, and larger multifocal perivascular and peribronchiolar mononuclear infiltrates (**Figure 3C**).

**Figure 4:**
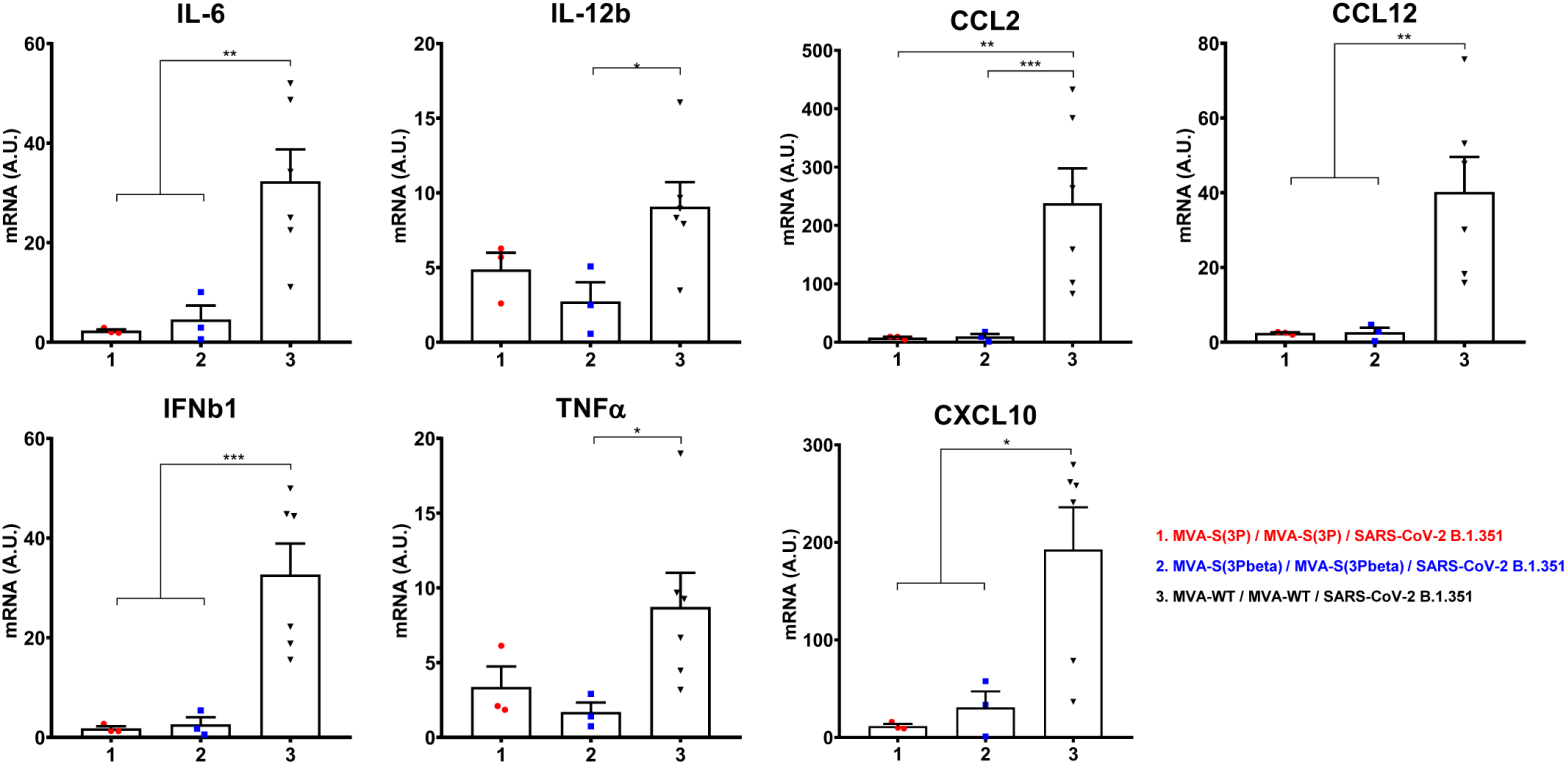
Vaccination with MVA-S(3P) or MVA-S(3Pbeta) diminished levels of proinflammatory cytokines in K18-hACE2 transgenic mice. mRNA levels of several cytokines/chemokines were detected by RT-qPCR in lungs obtained at 4 days postchallenge. Mean RNA levels (in A.U. normalized to uninfected mice) from duplicates of each sample and SEM of each group are represented. Ordinary one-way ANOVA of transformed data followed by Tukey’s multiple comparison test: *p < 0.033; **p < 0.002; ***p < 0.0002.

Furthermore, the impact of vaccination on the pro-inflammatory cytokine profile elicited in infected mice was analyzed at 4 days post-challenge by measuring the mRNA levels of key cytokines in lung homogenates by RT-qPCR (**Figure 4**). Vaccination with MVA-S(3P) or MVA-S(3Pbeta) elicited significant downregulation of IL-6, IL-12b, CCL2, CCL12, IFNβ1, TNF-α and CXCL10 mRNA levels, compared to control infected mice (**Figure 4**).

### MVA-S(3Pbeta) and MVA-S(3P) triggered, in K18-hACE2 transgenic mice, high titers of anti-S IgGs and neutralizing antibodies against Wuhan, beta and several VoCs

**Figure 5:**
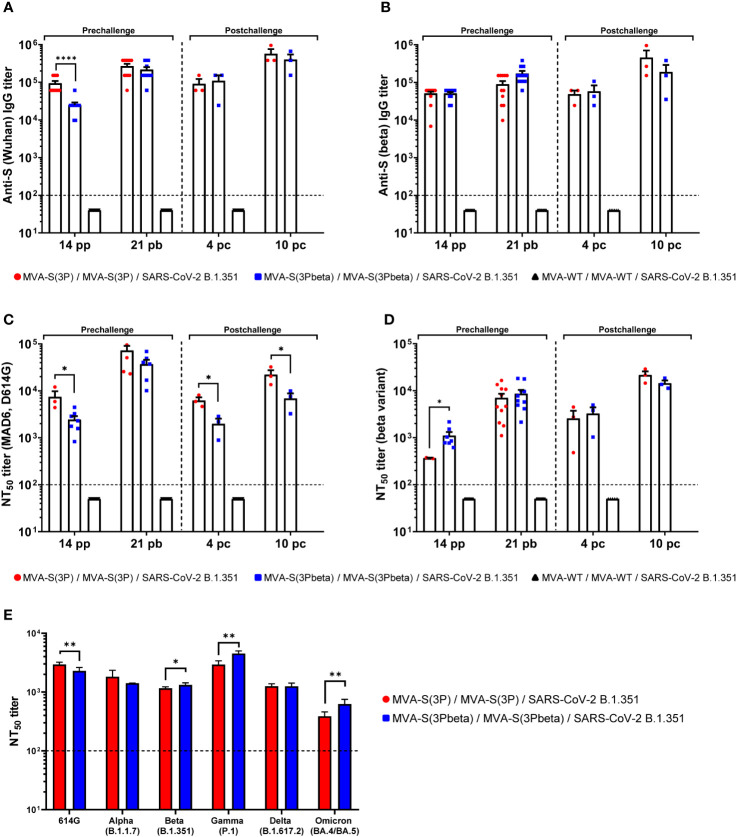
MVA-S(3P) and MVA-S(3Pbeta) vaccine candidates induced high levels of SARS-CoV-2-specific humoral immune responses in vaccinated and challenged K18-hACE2 transgenic mice. **(A, B)** Anti-S IgG titers against Wuhan **(A)** and beta variant **(B)** determined by ELISA in individual mouse serum samples from K18-hACE2 mice collected at day 14 post-prime (pp), 21 post-boost (pb), 4 and 10 postchallenge (pc). Mean endpoint titers of each sample from duplicates and SEM from each group are represented. Dashed line represents the limit of detection. Unpaired nonparametric Mann-Whitney test of transformed data: ****p < 0.0001. **(C, D)** SARS-CoV-2 neutralizing antibody titers against ancestral Wuhan strain (isolate MAD6, having D614G mutation) **(C)** and beta variant (B.1.351) **(D)**. NT_50_ titers were evaluated in individual mouse serum samples collected at day 14 pp, 21 pb, 4 and 10 pc) using a live virus MNT assay. Mean NT_50_ values of each sample from triplicates and SEM from each group are represented. Dotted line represented the limit of detection. Unpaired t-test of transformed data: *p < 0.033. **(E)** SARS-CoV-2 neutralizing antibody titers against several SARS-CoV-2 VoCs. NT_50_ titers were evaluated in pooled mouse serum samples collected at day 14 pp and 21 pb, using VSV-based pseudoparticles expressing the SARS-CoV-2 S protein of different VoCs. Mean NT_50_ values and 95% confidence intervals from triplicates of each pooled group sample are represented. The dashed line represents the limit of detection. Unpaired t-test with Welch’s correction of transformed data: *p < 0.033; **p < 0.002.

Since we have previously correlated antibody levels with protection against SARS-CoV-2 infection (35), we next analyzed in serum the SARS-CoV-2-specific humoral responses induced in transgenic K18-hACE2 mice vaccinated with MVA-S(3P) and MVA-S(3Pbeta), after vaccine immunization (days 14 post-prime and 21 post-boost) and post-challenge (days 4 and 10 post-challenge). At day 14 post-prime, MVA-S(3P) vaccinated mice elicited higher IgG titers against Wuhan S protein (**Figure 5A**), but similar IgG titers against beta S protein (**Figure 4B**) than MVA-S(3Pbeta) vaccinated mice. On day 21 post-boost and 4 and 10 post-challenge, the two vaccinated groups showed comparable anti-S IgG titers against Wuhan (**Figure 5A**) and beta variant (**Figure 5B**). Furthermore, in both vaccinated groups, S-specific IgG1, IgG2c, and IgG3 antibodies were elicited with IgG2c > IgG1 > IgG3 and IgG2c/IgG1 ratios above 1, indicating a Th1-like protective immune response (**Table 1**).

**Table 1:** Isotype analysis of anti-S IgG antibodies in transgenic K18-hACE2 mice immunized with MVA-S(3P) or MVA-S(3Pbeta).

Time points analyzed	Anti-S IgG1, IgG2c and IgG3 antibody titers and IgG2c/IgG1 ratio in transgenic K18-hACE2 vaccinated mice ^a^							
	MVA-S(3P) / MVA-S(3P) / SARS-CoV-2 B.1.351				MVA-S(3Pbeta) / MVA-S(3Pbeta) / SARS-CoV-2 B.1.351			
	IgG1	IgG2c	IgG3	IgG2c / IgG1	IgG1	IgG2c	IgG3	IgG2c / IgG1
Day 14 post-prime immunization	24414	152587	1562	6.25	9765	24414	250	2.5
Day 21 post-boost immunization	61035	381469	3906	6.25	61035	381469	1562	6.25
Day 4 post-challenge	24414	152587	9765	6.25	61035	152587	1562	2.5
Day 10 post-challenge	152587	953674	3906	6.25	152587	953674	1562	6.25

a Mean titer of IgG1, IgG2c and IgG3 antibody subclasses against SARS-CoV-2 S (Wuhan) protein, and IgG2c/IgG1 ratio, from duplicates of pooled sera samples obtained from the different immunization regimens studied.

Analysis of neutralizing antibody levels in serum revealed that, after the first immunization (day 14 post-prime) MVA-S(3P) induced significantly higher neutralizing antibody titers against live Wuhan than MVA-S(3Pbeta) (**Figure 5C**), while MVA-S(3Pbeta) induced significantly higher neutralizing antibody titers against live beta variant than MVA-S(3P) (**Figure 5D**). After two doses (day 21 post-boost) the levels of neutralizing antibodies against Wuhan and beta variant induced by both vaccine candidates enhanced, with a trend to higher NT_50_ titers against Wuhan in MVA-S(3P)-vaccinated mice and higher NT_50_ titers against beta variant in MVA-S(3Pbeta)-vaccinated mice (**Figure 5C, D**). After challenge (days 4 and 10 postchallenge), MVA-S(3P) induced significantly higher neutralizing antibody titers against live Wuhan than MVA-S(3Pbeta) (**Figure 5C**), while the levels of neutralising antibodies against beta variant induced by both vaccine candidates were comparable (**Figure 5D**).

Moreover, the analysis of neutralising antibodies against several VoCs in pooled sera from 21 days post-boost by using SARS-CoV-2 pseudotyped VSVs, showed that mice immunized with MVA-S(3P) induced significantly higher NT_50_ titers against Wuhan (D614G mutant) than MVA-S(3Pbeta), while mice immunized with MVA-S(3Pbeta) induced significantly higher NT_50_ titers against beta (B.1.351), gamma (P1), and Omicron BA.4/BA.5 VoCs (**Figure 5E**). NT_50_ titers against alpha (B.1.1.7) and delta (B.167.2) VoCs were similar between both vaccinated groups (**Figure 5E**).

### MVA-S(3Pbeta) and MVA-S(3P) induced local and systemic SARS-CoV-2 S-specific CD4^+^ and CD8^+^ T-cellular and humoral immune responses in immunized C57BL/6 mice

**Figure 6:**
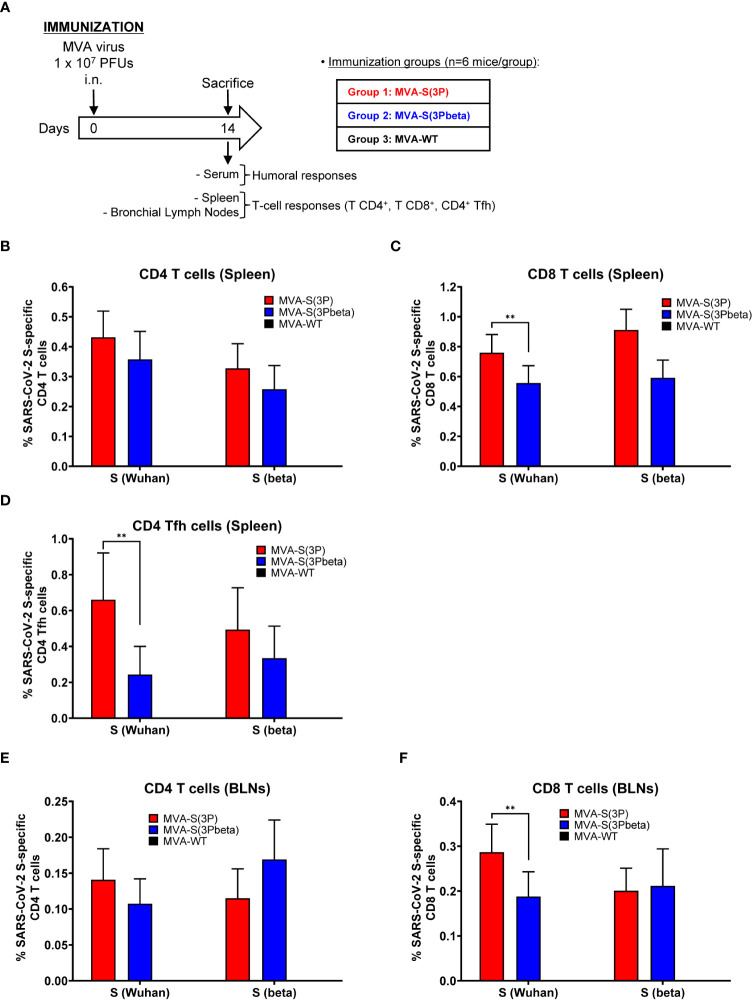
SARS-CoV-2-specific immunogenicity in C57BL/6 mice immunized with one IN dose of MVA-S(3P) and MVA-S(3Pbeta) vaccine candidates. **(A)** Immunogenicity study schedule. Groups of female C57BL/6 mice (n=6 per group; 6 to 8 weeks old) were slightly anesthetized and each mouse received one dose of 1 × 10^7^ PFUs of MVA-S(3P), or MVA-S(3Pbeta) by the IN route in 50 μl of PBS. Mice inoculated with nonrecombinant MVA-WT were used as a control group. At day 14 after the immunization, mice were euthanized and blood, spleens and BLNs from each mouse were collected. **(B–F)** SARS-CoV-2 S-specific T-cellular immune responses were evaluated in spleens **(B, C, D)**, and BLNs **(E, F).** Cell percentages were determined by ICS. **(B, C)** Magnitude of Wuhan strain and beta variant S-specific CD4^+^ **(B)** and CD8^+^ **(C)** T-cell immune responses in spleens. Percentages of CD4^+^ or CD8^+^ T cells expressing CD107a and/or producing IFN-γ and/or TNF-α and/or IL-2. **(D)** Magnitude of Wuhan strain and beta variant S-specific CD4^+^ Tfh cell responses in spleen. Percentages of CD4^+^ Tfh cells expressing CD40L and/or producing IFN-γ and/or IL-21. **(E, F)** Magnitude of Wuhan strain and beta variant S-specific CD4^+^ **(E)** and CD8^+^ **(F)** T-cell immune responses in BLNs. Percentages of CD4^+^ or CD8^+^ T cells expressing CD107a and/or producing IFN-γ and/or TNF-α and/or IL-2. **p < 0.002.

Since induction of cellular immune responses by SARS-CoV-2 vaccines is also an important marker for protective efficacy, we next assessed the SARS-CoV-2-specific T-cell immunogenicity induced in C57BL/6 mice (n=6/group) after one single IN dose of 1 × 10^7^ PFUs of MVA-S(3P), MVA-S(3Pbeta), or MVA-WT (as a negative control group). At 14 days postimmunization, mice were euthanized and SARS-CoV-2 S-specific T-cell immune responses were evaluated locally (in BLNs) or systemically (in spleen) (**Figure 6A**). Cells were stimulated *ex vivo* with S peptide pools, spanning the entire S protein from Wuhan strain or beta variant, and an ICS assay was performed to measure the induction of SARS-CoV-2 S-specific CD4^+^ and CD8^+^ T cells expressing CD107a, and/or secreting IFN-γ, TNF-α, and/or IL-2.

In spleen, MVA-S(3P) and MVA-S(3Pbeta) vaccinated mice elicited similar S-specific CD4^+^ T-cell immune responses against Wuhan strain and beta variant S peptide pools (**Figure 6B**). Regarding CD8^+^ T-cell responses, MVA-S(3P) vaccinated mice induced significantly higher magnitude against S peptide pools from Wuhan strain than MVA-S(3Pbeta) vaccinated mice, but both immunization groups exhibited similar beta variant S-specific CD8^+^ T-cell immune responses (**Figure 6C**). Moreover, similarly to CD8^+^ T-cell immune responses, MVA-S(3P) vaccinated mice elicited significantly higher Wuhan strain S-specific CD4^+^ T follicular helper (Tfh) immune responses (comprising CD4^+^ T cells expressing CD40L, and/or secreting IFN-γ, and/or IL-21), but of similar magnitude against beta variant (**Figure 6D**).

In BLNs, MVA-S(3P) and MVA-S(3Pbeta) vaccinated mice elicited similar S-specific CD4^+^ T-cell immune responses against Wuhan strain and beta variant S peptide pools (**Figure 6E**), while MVA-S(3P) vaccinated mice induced significantly higher Wuhan strain S-specific CD8^+^ T-cell immune responses than MVA-S(3Pbeta) vaccinated mice, but similar magnitude against beta variant (**Figure 6F**). Interestingly, both groups of vaccinated mice prompted higher magnitude of S-specific CD4^+^ and CD8^+^ T-cell immune responses in the spleens than in BLNs, being also the magnitude of CD8^+^ T cells higher than that of CD4^+^ T cells in spleen and BLNs (**Figure 6B, C, E, F**).

**Figure 7:**
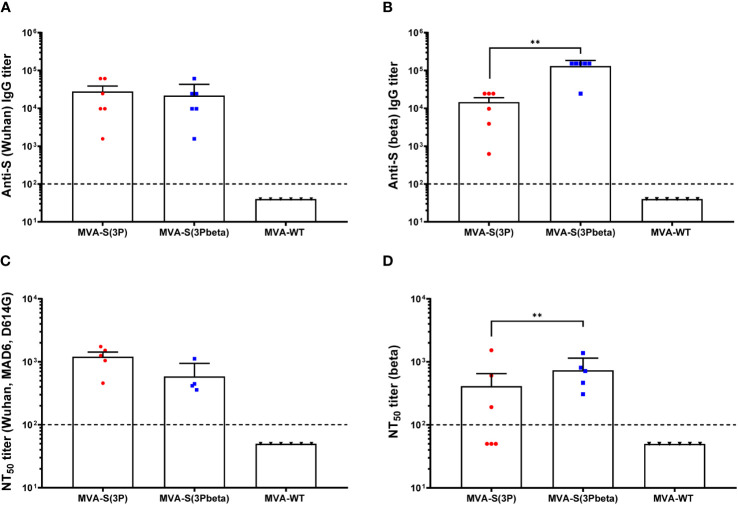
SARS-CoV-2-specific humoral immune responses elicited in C57BL/6 mice immunized with one IN dose of MVA-S(3P) and MVA-S(3Pbeta) vaccine candidates. SARS-CoV-2-specific humoral immune responses were evaluated in serum obtained at 14 days postimmunization. **(A, B)** Titers of binding IgG antibodies specific for the S protein from Wuhan strain **(A)** and beta variant **(B)**, determined by ELISA in individual mouse serum samples in duplicate. Mean values and SEM are represented. The dashed line represents the limit of detection. Unpaired nonparametric Mann-Whitney test of transformed data: **p < 0.002. **(C, D)** SARS-CoV-2 NT_50_ antibody titers determined in individual mouse serum samples by using a live virus MNT assay. Mean NT_50_ values and SEM against parental Wuhan strain virus (MAD6 isolate, containing D614G mutation) **(C)** or beta variant **(D)** are represented. The dashed line represents the limit of detection. Unpaired t-test of transformed data.

We also evaluated in serum samples the SARS-CoV-2-specific humoral immunogenicity induced in C57BL/6 mice after IN administration with one dose of 1 × 10^7^ PFUs of MVA-S(3P) and MVA-S(3Pbeta). At day 14 postimmunization, both immunization groups induced similarly high titers of total IgG antibodies against S of Wuhan strain (**Figure 7A**), but MVA-S(3Pbeta) elicited significantly higher titers of anti-S beta IgG antibodies than MVA-S(3P) (**Figure 7B**). By using a live MNT assay, we also detected high titers of SARS-CoV-2 neutralizing antibodies in serum samples from all vaccinated mice. MVA-S(3P) induced a trend towards higher NT_50_ titers against the parental Wuhan strain virus (MAD6 isolate, containing D614G mutation) than MVA-S(3Pbeta), but differences were not significant (**Figure 7C**). Similarly, MVA-S(3Pbeta) elicited a trend towards higher NT_50_ titers against the beta variant than MVA-S(3P), but differences were not significant (**Figure 7D**). MVA-WT were background, below the limit of detection; 1:100 dilution.

## Discussion

The current evolution of SARS-CoV-2 and the appearance of VoCs have raised concerns regarding the efficacy of the initially approved COVID-19 vaccines. These vaccines were designed and developed based on the antigenic characteristics of the original reference isolate, Wuhan-Hu-1. As the virus continues to evolve, there is a need to closely monitor the effectiveness of vaccines against newly emerging VoCs and consider the development of updated or modified vaccines to address any potential antigenic mismatch. The effectiveness of initially approved Wuhan-Hu-1-based COVID-19 vaccines against SARS-CoV-2 variants has been significantly reduced, especially against the Omicron variant (53, 54). Nevertheless, different studies have shown that some Wuhan-Hu-1-based COVID-19 vaccines can protect against SARS-CoV-2 VoC morbidity and mortality in different animal models, although with different degrees of infection protection (23–32). These results suggest that although approved COVID-19 vaccines can prevent hospitalization and death caused by VoCs, a variant-specific vaccine could ensure better protection against disease and beyond transmission. Therefore, recent research has focused on studying the ability of variant-adapted vaccine analogs to protect against ancestral SARS-CoV-2 and/or SARS-CoV-2 VoCs in different animal models (28, 30, 31, 55–57). We previously reported that immunization with one or two doses of an MVA-based vaccine candidate expressing a human codon-optimized full-length Wuhan-related SARS-CoV-2 S protein, MVA-S, induced robust titers of S- and RBD-binding IgG antibodies and neutralizing antibodies against parental SARS-CoV-2 and VoCs alpha, beta, gamma, delta, and to a lesser extent to omicron, either in mice (35–37), hamsters (39) or non-human primates (40). Moreover, a single dose of an optimized MVA-based vaccine candidate expressing a Wuhan-related full-length prefusion-stabilized SARS-CoV-2 S protein, MVA-S(3P), elicited in K18-hACE2 transgenic mice or in hamsters higher titers of S- and RBD-specific IgGs and neutralizing antibodies against different SARS-CoV-2 VoCs than MVA-S (36, 37, 41). To define the immune contribution of a VoC to control SARS-CoV-2 infections, in this study we generated a novel beta (B.1.351)-adapted vaccine analog of MVA-S(3P), termed MVA-S(3Pbeta). Afterwards, we studied in transgenic K18-hACE2 mice the degree of protection of both MVA-S(3P) and MVA-S(3Pbeta) vaccine candidates against a lethal challenge with SARS-CoV-2 beta variant, and analyzed the correlation with the SARS-CoV-2-specific induced humoral immune responses. Concurrently, we investigated the T-cell immunogenicity induced in C57BL/6 mice after one IN administration of these two vaccine candidates.

The efficacy study showed that immunization with two IM doses of both vaccine candidates prevented death and body weight loss in K18-hACE2 mice challenged with SARS-CoV-2 B.1.351 variant and significantly reduced the levels of SARS-CoV-2 genomic and subgenomic mRNA in lung and BAL samples, as well as the levels of key proinflammatory cytokines. Importantly, no live infectious virus was detected in the lung and BAL samples of vaccinated mice compared to the MVA-WT control group. These results are similar to those recently reported by other MVA-based vaccine candidates expressing ancestral Wuhan S protein that prevented death and body weight loss in mice challenged with a SARS-CoV-2 beta variant (58, 59). Our findings demonstrate the efficacy of MVA-S(3P) and MVA-S(3Pbeta) vaccine candidates in preventing morbidity and mortality in the context of beta-variant infections, and supporting the clinical evaluation of original or analogous variant-adapted vaccine candidates based on recombinant MVA-expressing prefusion-stabilized SARS-CoV-2 S protein.

Both vaccine candidates were able to induce in serum high anti-S IgG and neutralizing titers against ancestral SARS-CoV-2, beta variant and other VoCs. Small significant differences in strain-specific anti-S IgG and neutralizing titers were observed in the efficacy study in K18-hACE2 mice after the immunizations and after SARS-CoV-2 beta variant challenge. In general, mice immunized with MVA-S(3P) induced significantly higher anti-S IgG and NT_50_ antibody titers against Wuhan than MVA-S(3Pbeta) and mice immunized with MVA-S(3Pbeta) elicited significantly higher anti-S IgG and NT_50_ antibody titers against beta variant than MVA-S(3P). Similarly, in the immunogenicity study in C57BL/6 mice immunized with one IN dose of MVA-S(3P) or MVA-S(3Pbeta), we also detected significantly higher beta variant anti-S IgG titers in mice immunized with MVA-S(3Pbeta) than in those with MVA-S(3P), and a trend to higher NT_50_ antibody titers. The primary reason for these small differences appeared to be the level of genetic similarity (homology) between the vaccine antigen and the targeted antibody responses, with the response being more robust when the antigen matches the specific variant. Other studies have also observed strain-specific differential neutralizing responses when comparing the neutralizing capacity induced by Wuhan-Hu-1-specific vaccines with that of variant-adapted vaccine analogs (30, 53, 56, 57). For example, in Syrian hamsters vaccinated with two IM doses of a multi-antigenic COVID-19 Wuhan-Hu-1-S-specific vaccine candidate based on a synthetic MVA (sMVA) vector or its beta-adapted vaccine analog, it was reported a similar neutralizing activity against the ancestral Wuhan virus in both immunization groups but a higher neutralizing activity against beta and delta variants in hamsters vaccinated with the beta-analog compared with hamsters vaccinated with the COVID-19 Wuhan-Hu-1-specific vaccine candidate (30). Moreover, a significantly higher neutralizing activity against the ancestral Wuhan virus but significantly lower neutralizing titers against the beta and delta variants has been reported in mice vaccinated with two IM doses of a recombinant COVID-19 Wuhan-Hu-1-S-specific vaccine candidate based on MVA (rMVA) (56). Furthermore, it has been also stated a significant decrease in live-virus neutralization titers against SARS-CoV-2 beta variant in comparison with the ancestral Wuhan strain in mice vaccinated with an optimized MVA-based SARS-CoV-2 vaccine expressing Wuhan-Hu-1-S with the furin cleavage site inactivated and N proteins (57). In addition, it has been described significant distinct neutralizing responses in mice and hamsters vaccinated with 1 μg of COVID-19 Wuhan-Hu-1-S-specific RNA and beta-variant analog vaccine candidates when comparing homologous to heterologous infectious virus: a significant reduction in neutralization activity against the beta variant in animals vaccinated with their Wuhan-Hu-1-specific vaccine candidate and against ancestral virus in animals vaccinated with its beta-variant analog (53). These outcomes are also consistent with prior investigations showing diminished neutralization against beta (B.1.351) variant of sera from Wuhan-infected convalescents and Wuhan-based first-generation COVID-19 vaccines in vaccinees (7, 8, 60). In contrast, different results were observed in BALB/c mice after one IM dose of an ChAdOx1 Wuhan-Hu-1-specific vaccine or a beta-adapted vaccine analog expressing a non-stabilized S protein, where comparable levels of neutralizing capacity against both variants were observed (24). Further research is needed to understand the role of the antigenic variability between variants, and its relevance in humans.

In the efficacy study involving K18-hACE2 mice, after two immunization doses with both vaccine candidates, the levels of anti-S IgG antibodies and NT_50_ antibody titers against both the Wuhan strain and the beta variant were significantly enhanced and comparable between the two vaccines. In addition, the analysis of neutralizing antibodies against several VoCs in pooled sera by using SARS-CoV-2 pseudotyped VSVs, showed that mice immunized with two IM doses of MVA-S(3P) or MVA-S(3Pbeta) elicited cross-neutralizing antibodies against other VoCs such as alpha (B.1.1.7), delta (B.167.2), gamma (P1), and Omicron BA.4/BA.5. This result confirms that repetitive booster immunizations with vaccines based on the ancestral Wuhan strain or vaccine analogs can significantly improve immune responses against SARS-CoV-2, including VoCs. Similar findings have been previously reported with approved COVID-19 vaccines (13, 61). Because of the potent humoral responses elicited by the MVA-S(3P) and MVA-S(3Pbeta) vaccine candidates, both immunization groups showed full efficacy in protecting K18-hACE2 mice from mortality, weight loss, lower respiratory tract infection, and lung pathology following a lethal challenge with the SARS-CoV-2 beta variant, consistently demonstrating that both vaccine candidates elicited broadly cross-protective SARS-CoV-2 immunity. This indicates that a full antigenic match between the vaccine and the challenged virus is not needed for protection of the lower respiratory tract. These results further support the clinical evaluation of MVA-S(3P) or MVA-S(3Pbeta) vaccine candidates as potential booster doses in COVID-19 vaccination regimens.

As in addition to antibody responses, protection against SARS-CoV-2 infection by vaccines also correlates with induction of T-cell immune responses, we analyzed the impact of one single IN administration of MVA-S(3P) or MVA-S(3Pbeta) in the activation of CD4^+^ and CD8^+^ T cells. We used a single dose and IN route to better observe the potential differences between both vaccine candidates (36). Systemic (in spleen) and local (in BLNs) S-specific T-cell responses follow the same pattern, with MVA-S(3P) and MVA-S(3Pbeta) vaccinated mice eliciting similar S-specific CD4^+^ T-cell immune responses against the Wuhan strain than the beta variant. However, for CD8^+^ T-cell response, MVA-S(3P) vaccinated mice induced significantly higher CD8^+^ T-cell immune responses specific to the Wuhan strain compared to MVA-S(3Pbeta) vaccinated mice, while both groups exhibited a similar magnitude of response against the beta variant. Once again, these subtle yet significant differences in strain-specific T-cell responses indicate a distinction in epitope specificity and antigenicity of the S antigens. Nevertheless, these differences may be restricted to one dose immunization as it was previously shown that vaccine-induced T cells targeting the ancestral S protein demonstrate minimal reductions after booster vaccination in frequency and magnitude against the beta variant and other VoCs (62–64). Besides the essential role of T cells in identifying and eliminating virus-infected cells, specific T-cell responses also support B cell and antibody generation via CD4^+^ Tfh cells, which are instrumental to limit SARS-CoV-2 infection (65). Here, we find that one IN immunization with MVA-S(3P) or MVA-S(3Pbeta) was able to induce S-specific CD4^+^ Tfh cells expressing CD40L, and/or secreting IFN-γ, and/or IL-21, confirming our previous observation (36). Differences in epitope specificity and antigenicity of the MVA-S(3P) and MVA-S(3Pbeta) antigens may explain the higher Wuhan S-specific CD4^+^ Tfh magnitude observed in mice vaccinated with MVA-S(3P) compared with those vaccinated with MVA-S(3Pbeta).

In conclusion, our vaccine platform was successfully adapted to target emerging VoCs, and our results support the sustained development of the MVA vaccine platform for COVID- 19 and the clinical evaluation of MVA-S(3P) and MVA-S(3Pbeta) as booster vaccines to diversify and potentially increase the recognition of highly mutated VoC.

## Data availability statement

The raw data supporting the conclusions of this article will be made available by the authors, without undue reservation.

## Ethics statement

The animal study was approved by the Ethical Committees of Animal Experimentation (CEEA) of the CNB-CSIC, CISA-INIA-CSIC, and the Division of Animal Protection of the Comunidad de Madrid (PROEX 49/20, 169.4/20, and 161.5/20). Animal procedures conformed with international guidelines and with Spanish law under the Royal Decree (RD) 53/2013. The study was conducted in accordance with the local legislation and institutional requirements.

## Author contributions

PP: Conceptualization, Data curation, Formal Analysis, Investigation, Methodology, Supervision, Validation, Visualization, Writing – original draft, Writing – review & editing. GA: Data curation, Formal Analysis, Investigation, Methodology, Validation, Visualization, Writing – review & editing. DA: Data curation, Formal Analysis, Investigation, Methodology, Validation, Visualization, Writing – review & editing. SF: Investigation, Methodology, Writing – review & editing. CS-C: Investigation, Methodology, Writing – review & editing. PS-C: Investigation, Methodology, Writing – review & editing. JL: Investigation, Methodology, Writing – review & editing. RD: Funding acquisition, Resources, Writing – review & editing. JC: Resources, Writing – review & editing. ME: Conceptualization, Funding acquisition, Writing – review & editing. JG-A: Conceptualization, Data curation, Formal Analysis, Funding acquisition, Investigation, Methodology, Supervision, Validation, Visualization, Writing – original draft, Writing – review & editing.
